# Risk factors for acquisition of colistin-resistant *Klebsiella pneumoniae* and expansion of a colistin-resistant ST307 epidemic clone in hospitals in Marseille, France, 2014 to 2017

**DOI:** 10.2807/1560-7917.ES.2021.26.21.2000022

**Published:** 2021-05-27

**Authors:** Sophie Alexandra Baron, Nadim Cassir, Mouna Hamel, Linda Hadjadj, Nadia Saidani, Gregory Dubourg, Jean-Marc Rolain

**Affiliations:** 1 Aix Marseille Univ, IRD, APHM, MEPHI, Faculté de Médecine et de Pharmacie, Marseille, France; 2 IHU Méditerranée Infection, Faculté de Médecine et de Pharmacie, Marseille, France; 3 Aix Marseille Univ, IRD, AP-HM, SSA, VITROME, Marseille, France

**Keywords:** colistin resistance, risk factors, outbreak, *Klebsiella pneumoniae*, ST307

## Abstract

**Background:**

France is a low prevalence country for colistin resistance. Molecular and epidemiological events contributing to the emergence of resistance to colistin, one of the 'last-resort' antibiotics to treat multidrug-resistant Gram-negative infections, are important to investigate.

**Aim:**

This retrospective (2014 to 2017) observational study aimed to identify risk factors associated with acquisition of colistin-resistant *Klebsiella pneumoniae* (CRKP) in hospitals in Marseille, France, and to molecularly characterise clinical isolates.

**Methods:**

To identify risk factors for CRKP, a matched-case–control (1:2) study was performed in two groups of patients with CRKP or colistin-susceptible *K. pneumoniae* respectively. Whole-genome-sequences (WGS) of CRKP were compared with 6,412 *K. pneumoniae* genomes available at the National Center for Biotechnology Information (NCBI).

**Results:**

Multivariate analysis identified male sex and contact with a patient carrying a CRKP as significant independent factors (p < 0.05) for CRKP acquisition, but not colistin administration. WGS of nine of 14 CRKP clinical isolates belonged to the same sequence type (ST)307. These isolates were from patients who had been hospitalised in the same wards, suggesting an outbreak. Comparison of the corresponding strains’ WGS to *K. pneumoniae* genomes in NCBI revealed that in chromosomal genes likely playing a role in colistin resistance, a subset of five specific mutations were significantly associated with ST307 (p < 0.001).

**Conclusion:**

A ST307 CRKP clone was identified in this study, with specific chromosomal mutations in genes potentially implicated in colistin resistance. ST307 might have a propensity to be or become resistant to colistin, however confirming this requires further investigations.

## Introduction

Since the mid-2000s, carbapenem resistance has led to the revival of colistin, a polymyxin, as a last resort antibiotic [1] to treat Gram-negative bacterial infections [2], including carbapenemase-producing *Klebsiella pneumoniae* [2]. In this regard, the recent emergence of colistin resistance in humans and animals, particularly via the mobile colistin resistance gene (*mcr*) is concerning [1]. For *K. pneumoniae,* a study between 2015 and 2017, concerning 18 European countries estimated an overall colistin resistance rate of 5.4% [3]. Other surveillance activities in Europe found resistance to polymyxins in this species varying from 2.6% in carbapenem-susceptible strains to 31.9% in carbapenem-resistant ones [4].

In the literature, previous use of colistin has been reported as a risk factor for colistin resistance. Nevertheless, colistin-resistant bacteria have also been isolated from people who had not previously been treated with this drug [5] and, for* K. pneumoniae,* some investigations point to other risk factors in patients, such as previous carbapenemase-producing *K. pneumoniae* colonisation, corticosteroid administration and prior hospitalisations. The aforementioned risk factors were mainly assessed in Greece [6] and Italy [7], where the rate of colistin resistance reached 30%, which is much higher than in France [8]. In our hospitals in Marseille, France, susceptibility to colistin is only tested in certain circumstances (see methods). As a result, the prevalence of colistin resistance cannot be estimated. Moreover, the mechanisms of colistin resistance as well as the risk factors associated with the acquisition of colistin-resistant organisms have thus far not been assessed in our setting.

The *K. pneumoniae* sequence type (ST)307 was first reported in a clinical case in 2009 in Pakistan but subsequent findings indicated that this ST might have already emerged in the mid-1990s [9,10]. ST307 has been involved in local hospital outbreaks in Africa, the Americas, Asia and Europe [9]. It is frequently associated with multidrug resistance, especially with CTX-M-15 extended-spectrum beta-lactamase (ESBL) and carbapenemase (*K. pneumoniae* carbapenemase; KPC) and it has even replaced the multidrug-resistant international ST258 in some countries such as Canada and Italy [11]. In 2018, a whole genome sequencing (WGS) and epidemiologic analysis of KPC-*Klebsiella pneumoniae* isolates in France found the emergence of several high-risk clones in the hospital environment including ST307 [12]. Among studies focusing on ST307 multidrug resistance however, few have investigated colistin resistance [10,13,14]. On the other hand, of *K. pneumoniae* STs reported to be colistin-resistant, those belonging to clonal complexes (CC)258 (ST258, ST512 and ST11) and CC15 have most frequently been identified [10].

The aim of our work was to find colistin-resistant *K. pneumoniae* (CRKP) isolated from patients hospitalised in Marseille university hospitals between February 2014 and May 2017, to molecularly characterise the strains, and to determine risk factors associated with CRKP acquisition. Because strains of ST307 dominated among the CRKP identified, these were also the object of further focus and epidemiological investigations.

## Methods

### Collection of colistin-resistant *Klebsiella pneumoniae* strains and microbiological procedures

This retrospective study was conducted on patients who had been hospitalised in Marseille university hospitals, France, from February 2014 to August 2017. These four hospitals (Timone, Conception, North, and Sainte-Marguerite hospitals) have a total of 3,700 beds.

Our laboratory carries out the microbiological analysis of specimens collected in the four hospitals. Colistin susceptibility testing is performed on *K. pneumoniae* isolates that are susceptible to less than three families of antibiotics included in our routine antibiotic testing panel, or upon specific request. We selected all *K. pneumoniae* strains (one per patient) for which a colistin minimum inhibitory concentration (MIC) was > 2 µg/mL (performed by E-test or microdilution method) according to the European Committee on Antimicrobial Susceptibility Testing (EUCAST) recommendations [15]. Antimicrobial susceptibility testing for other antibiotics was done according both the EUCAST and Comité de l’Antibiogramme – Société Française de Microbiologie (CA-SFM) recommendations using the disk diffusion method on Mueller–Hinton (MH) agar (BioMérieux, Marcy l’Etoile, France) enriched with commercial antibiotic disks, and/or E-test (Biomérieux) as previously described [16].

### Study of risk factors for colistin-resistant *Klebsiella pneumoniae* acquisition

Cases were defined as patients for whom a CRKP had been isolated at least once in one sample, thus including patients colonised and/or infected with CRKP. Patients' clinical histories were obtained from their electronic medical records. Each case was included in the study only once, i.e. at the time of the first CRKP isolation. In order to assess the risk factors for CRKP carriage, cases were compared to control patients colonised or infected with colistin-susceptible *K. pneumoniae* (CSKP) strains isolated during the same study period. In this case–control analysis, controls were matched to cases (2:1) by age, type of ward (medical, surgical or intensive care) and type of sample. Comorbidities/conditions analysed were as follows: organ transplant recipient, chronic renal disease, haematologic or solid organ cancer (excluded if remission > 5 years), chronic pulmonary disease, diabetes mellitus, splenectomy, cerebrovascular disease, liver cirrhosis and heart disease. Modified Charlson scores ≥ 3 [17] were calculated and we considered several aspects of the patient’s recent medical history such as: previous surgery (< 1 year prior to the current study), previous antibiotic therapy (< 3 months prior), recent history of bacterial infection (< 1 month prior), previous isolation of an ESBL or a carbapenemase-producing bacteria, use of mechanical ventilation, haemodialysis, urinary catheter and central venous catheter. The final diagnosis (e.g. infection or colonisation) defined by the practitioner in his final report was also retrieved.

### Statistical analysis

The selected variables were compared by the chi-squared test, Fisher’s exact test and the Student’s t-test as appropriate. Univariate analysis was two-sided and a significant p value was chosen at p < 0.05. Multivariate analysis was performed by conditional logistic regression, and only the risk factors with a p value < 0.05 in univariate analysis were used for the multivariate analysis. Statistical analysis was performed using the SPSS software (SPSS Inc, Chicago, Illinois, United States (US)).

### Genomic study

#### Whole genome sequencing, bioinformatical analysis and clonal relationship

Genomic DNAs of *K. pneumoniae* strains were sequenced with the MiSeq Technology (Illumina, San Diego, US) with a 2 × 250 paired-end run strategy using the Nextera Mate Pair sample prep kit (Illumina). Genome assembly was done using the A5-miseq software (http://sourceforge.net/projects/ngopt/) and annotated with Prokka (https://github.com/tseemann/prokka). Plasmid replicons, virulence and resistance genes were sought with Abricate. Genome files were deposited in the National Center for Biotechnology Information (NCBI) database under the Bioproject number PRJNA520974 (Supplementary Table S1).

#### Clonal relationship study

Multilocus sequence typing (MLST) analysis was performed in silico to determine the ST of collected strains using the Bacterial Isolate Genome Sequence Database (BIGSdb) database (http://bigsdb.pasteur.fr/klebsiella/klebsiella.html). The *wzi* locus type was also identified with information from the BIGsdb database. The pangenome of *K. pneumoniae* isolates was determined using Roary [18]. We used in a first step the core gene alignment output file to construct the maximum likelihood phylogenetic tree using the RaXML software. In a second step, we performed a genome comparison based on single nucleotide polymorphism (SNP)s variants using the CSI Phylogeny (version 1.4) available on the Center for Genomic Epidemiology website (https://cge.cbs.dtu.dk/services/CSIPhylogeny). An average of 10 SNP (range: 0–30) was chosen as cut-off to define isolates of a same clone as previously described [19].

#### In silico mutations analysis

Sequences of proteins involved in colistin resistance were compared with the colistin-susceptible (MIC = 2 mg/L) [20] reference strain *K. pneumoniae* MGH78578 (GenBank accession number: CP000647). The deleterious effect of detected mutations on the function of proteins was investigated using Protein Variation Effect Analyzer (PROVEAN; http://provean.jcvi.org/index.php). Moreover, we investigated the frequency of the mutations found in *PmrA/B*, *CrrA/B* [20] and *AcrR/S *[21-23] in all ST307 among the 6,412 genomes of *K. pneumoniae* available on NCBI (last upgrade April 2019) by Basic Local Alignment Search Tool (BLAST) protein (blastp) using as threshold expect (E)-value 10e^−5^. Next we performed MLST using PubMLST typing schemes by local BLAST on all *K. pneumoniae* genomes available in NCBI to compare the frequency of different STs in this database.

### Ethical statement

This study was qualified as an internal study not involving the human person, as it was conducted on the basis of data collected as part of the individual therapeutic or medical follow-up of patients, by the personnel providing this follow-up and for their exclusive use. It was approved by the sites' institutional review boards with a waiver of informed consent and registered under number RGPD/Ap-Hm 2020–02.

## Results

From February 2014 to August 2017, 22 patients carried a CRKP isolate among the 5,304 patients who had at least one *K. pneumoniae* isolated in our laboratory. During this period, 374 determinations of colistin MIC were performed. The median age of the 22 patients was 69 years (range: 53–76) and 18 were males (sex ratio = 4.5). Four strains were isolated in 2014, eight in 2015, seven in 2016 and three in 2017. Strains were recovered from five respiratory, six urine, and five stool samples as well as one blood culture, one bone biopsy, two liquid punctures and two cutaneous swabs. Colistin MIC varied from 4 to > 64 µg/mL (Supplementary Tables S2 and S3).

### Risk factors for colistin resistance acquisition

The 22 CRKP cases were matched by age, type of ward and sample type with 44 controls carrying a CSKP isolated during the same study period. Univariate analysis of the two groups identified as significant risk factors male sex, length of hospital stay, recent bacterial infection, previous isolation of an ESBL or a carbapenemase producing bacteria and contact with cases carrying a CRKP (Table 1). Finally, previous antibiotic therapy, especially by fluoroquinolones, aminoglycosides or carbapenems was associated with an increased risk to acquire CRKP.

**Table 1:** Univariate analysis of characteristics potentially presenting a risk factor for colistin-resistant *Klebsiella pneumoniae* acquisition, Marseille, France, 2014–2017 (n = 66 patients)

Characteristics		CRKP n = 22	CSKP n = 44	p value
Age of patients in years; median (range)		69 (53–76)	66 (53–75)	NS
Number of patients of male sex		18	19	**0.004**
Length of hospital stay in days; mean (standard deviation)		60.1 (56.6)	44.7 (40.3)	**< 0.001**
Delay before first positive sample in days; mean (standard deviation)		21.7 (22.1)	13.2 (17.2)	0.151
Recent travel; number of patients (%)		2 (9)	3 (7)	NS
Type of ward; number of patients (%)	Intensive care unit	15 (68)	30 (68)	NS
	Surgery services	3 (14)	6 (14)	NS
	Medical services	3 (14)	6 (14)	NS
	Neonatal intensive care unit	1 (5)	2 (5)	NS
Type of sample; number of patients (%)	Respiratory	6 (27)	13 (30)	0.848
	Urine	6 (27)	18 (41)	0.279
	Blood	1 (5)	4 (9)	0.911
	Bone	1 (5)	2 (5)	NS
	Liquid puncture	2 (9)	4 (9)	NS
	Cutaneous swab	2 (9)	6 (14)	0.923
	Rectal swab	7 (32)	14 (32)	NS
Comorbidies; number of patients (%)	Solid organ cancer	8 (36)	7 (16)	0.062
	Haematologic cancer	1 (5)	3 (7)	NS
	Cerebrovascular disease	0 (0)	6 (14)	0.167
	Liver disease	1 (5)	2 (5)	NS
	Chronic renal failure	2 (9)	7 (16)	0.706
	Chronic pulmonary disease	2 (9)	5 (11)	NS
	Diabetes mellitus	5 (23)	13 (30)	0.770
	Cardiovascular disease	7 (32)	12 (27)	0.701
	Solid organ transplantation	0 (0)	3 (7)	0.545
	Charlson score ≥ 3	16 (73)	32 (73)	NS
History; number of patients (%)	Previous surgery	10 (45)	15 (34)	0.370
	Mechanical ventilation	14 (64)	20 (45)	0.164
	Central venous catheter	16 (73)	32 (73)	NS
	Intravenous home therapy	3 (14)	4 (9)	0.678
	Urinary catheter	16 (73)	25 (57)	0.209
	Haemodialysis	4 (18)	3 (7)	0.210
	Recent bacterial infection	12 (55)	13 (30)	**0.048**
	Previous isolation of an ESBL-producing bacteria	3 (14)	7 (16)	NS
	Previous isolation of a carbapenemase producing bacteria	11 (50)	6 (14)	**0.006**
	Contact with patient carrying a CRKP	14 (64)	12 (27)	**0.004**
Previous treatment; number of patients (%)	Amoxicillin + clavulanic acid	4 (18)	9 (20)	NS
	Piperacillin + clavulanic acid	9 (41)	17 (39)	0.859
	Third generation cephalosporin	13 (59)	15 (34)	0.053
	Carbapenems (imipenem, ertapenem or meropenem)	9 (41)	7 (16)	**0.025**
	Fluoroquinolones	11 (50)	21 (48)	**0.025**
	Aminoglycosides	15 (68)	15 (34)	**0.009**
	Colistin	3 (14)	2 (5)	0.323

CRKP: colistin-resistant *Klebsiella pneumoniae*; CSKP: colistin-susceptible *Klebsiella pneumoniae*; ESBL: extended-spectrum beta-lactamase; NS: non-significant p value.

In the multivariate analysis, male sex (p < 0.05) and contact with a case carrying a CRKP (p < 0.01) remained the only independent factors for CRKP acquisition (Table 2). 

**Table 2:** Independent risk factors for acquisition of colistin-resistant *Klebsiella pneumoniae* found through multivariate analysis, Marseille, France, 2014–2017 (n = 66 patients)

Risk factors	OR (95% CI)	p value
Male sex	2.5 (1.5–12.5)	0*.*023
Contact with patient carrying a CRKP	3.7 (1.8–17.0)	0*.*014

CI: confidence interval; CRKP: colistin-resistant *Klebsiella pneum*oniae; OR: odds ratio.

In our study, only five patients had previously received colistin. Three cases with CRKP received at least intravenous colistin, while two patients with CSKP received only aerosolised colistin (Supplementary Table S4).

Twelve cases among the 22 in the CRKP group were infected vs 21 of 44 patients in the control group (p = 0.862). Mortality rate reached 18% (4/22) in the CRKP group vs 25% (11/44) in the CSKP group (p = 0.770). In the CRKP group, one death was attributable to *K. pneumoniae* infection.

### Clinical and biological features of cases with acquired colistin-resistant *Klebsiella pneumoniae*

We compared cases with a CRKP infection to those who were just colonised by CRKP. The length of stay in hospital (37 vs 88 days; p = 0.025) as well as the delay before the first positive CRKP sample (14 vs 31 days; p = 0.0385) were significantly shorter in the infected group than in the colonised group. However, while the delay in the colonised group was based on 10 cases, only five of them had tested negative for CRKP at admission. The remainder had not been tested at that time, but were nevertheless used to estimate delay, under the assumption that testing was not performed, due to absence of risk factors requiring this. No other significant difference was observed between the two groups. No specific ST was associated with infection or colonisation.

### Whole genome sequencing study and resistome

As this work was a retrospective study and all laboratory strains are not permanently stored, only 14 of the 22 CRKP isolates from the microbiology laboratory were available for WGS analysis. Genomes sizes ranged from 5,518,326 bp to 5,912,443 bp with a percentage of GC bases ranging from 56.3 to 57.3%. Genomes were assembled in 74 to 603 contigs with a 19 to 55 coverage. The main features of the different genomes are reported in Supplementary Tables S2 and S3.

Of 14 colistin-resistant *K. pneumoniae* strains, nine belonged to ST307, followed by two to ST15, two to ST101 and one to ST322. Antibiotic susceptibility testing was performed on the 14 sequenced strains (Supplementary Tables S2 and S3).

**Figure 1:**
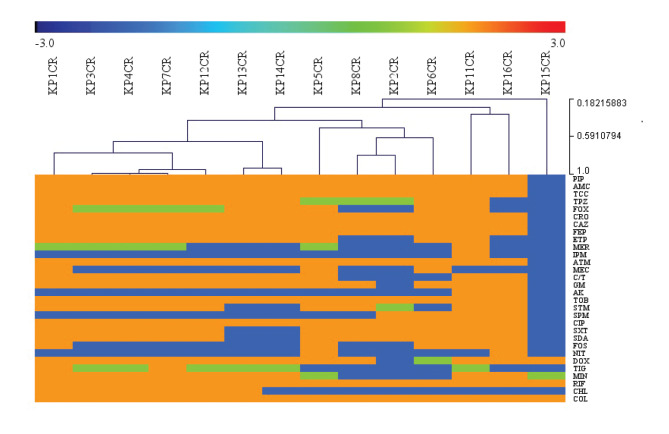
Hierarchical tree of antimicrobial susceptibility testing results of sequenced colistin-resistant *Klebsiella pneumoniae* isolates, Marseille, France, 2014–2017 (n = 14 isolates)

Resistance rate to each antibiotic is presented in Figure 1. The isolates were mostly susceptible to imipenem (13 isolates), nitrofurantoin (12 isolates), amikacin (12 isolates), minocycline (11 isolates), mecillinam (11 isolates), fosfomycin (nine isolates), chloramphenicol (eight isolates) and tigecycline (six isolates) (Figure 1). However, the combination of ceftolozane and tazobactam was active on only four isolates.

AK: amikacin; AMC: amoxicillin + clavulanic acid; ATM: aztreonam; CAZ: ceftazidime; CHL: chloramphenicol; CIP: ciprofloxacin; COL: colistin; CRO: ceftriaxone; C/T: ceftolozane + tazobactam; DOX: doxycyline; ETP: ertapenem; FEP: cefepime; FOS: fosfomycin; FOX: cefoxitin; GM: gentamicin; IPM: imipenem; MEC: mecillinam; MER: meropenem; MIN: minocycline; NIT: nitrofurantoin; PIP: piperacillin; RIF: rifampicin; SDA: sulfadiazine; SPM: spectinomycin; STM: streptomycin; SXT: trimethoprim + sulfamethoxazole; TCC: ticarcillin + clavulanic acid; TIG: tigecycline; TOB: tobramycin; TPZ: piperacillin + tazobactam.

The colistin resistant (CR) *Klebsiella pneumoniae* (KP) isolates/strains are listed above the diagram and in each column under them, the susceptibility profile to specific antibiotics, which are listed to the right, is depicted. Orange indicates that the strain is classified as resistant to an antibiotic, green that the strain is categorised as intermediate and blue that the strain is classified as susceptible.

The nine ST307 CRKP carried *bla_OXA-1_, bla_SHV-28_, aac(3)-IIa, aac(6’)Ib-cr, qnrB1, tetA*, and *catB4* genes (Supplementary Tables S2 and S3). All but two (KP5CR and KP8CR) of these strains carried a *bla_OXA-48_*. The two ST15 CRKP (KP2CR and KP6CR on Figure 1) were positive for *bla_OXA-1_, bla_SHV-28_, bla_TEM-1_, bla_CTX-M-15_, aac(6’)Ib-cr, aadA2, aph(3’)-Ia, sulI, dfrA* and *catB4.* One ST15 strain (KP6CR) was also carrying *bla_DHA-1_, aac(3)-IIa, qnrB1, qnrB4, mph(A)* genes. The two CRKP ST101 (KP11CR and KP16CR) had a *bla_OXA-1_, bla_OXA-9_, bla_SHV-15_, bla_CTX-M-15_, aac(3)-IIa, dfrA, tetD* and *catB4* genes. The strain KP11CR also carried *aac(6’)Ib, bla_OXA-48_* genes. The strain KP16CR was also positive for *bla_TEM-1_, sulII, tetA*. Finally, the ST322 strain (KP15CR), which was susceptible to all antibiotic but cyclins, was carrying a *tetB* and a *bla_SHV-11_* gene.

### Spread of an epidemic ST307 colistin-resistant *Klebsiella pneumoniae* in intensive care units

**Figure 2:**
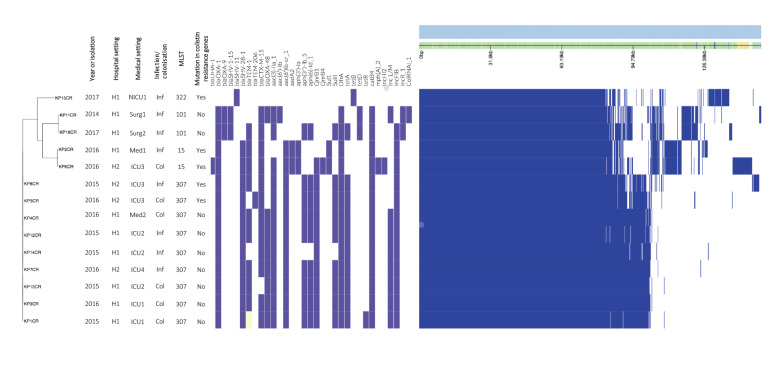
Phylogenetic tree based on core genome sequence alignment of colistin-resistant* Klebsiella pneumoniae*, Marseille, France, 2014–2017 (n = 14 sequences)

CR: colistin-resistant; ICU: intensive care unit; H: hospital; Inf: infection; KP:* Klebsiella pneumoniae*; Med: medical ward; MLST: multilocus sequence typing; Surg: surgery ward.

The pangenome of the 14 isolates recovered 7,897 genes, including 4,136 genes belonging to the core genome and 3,761 to the accessory genome (Figure 2). A phylogenetic tree based on the core genome showed that the nine ST307 strains were closely related (2–114 SNPs) and suggested that they could possibly belong to the same clone.

The following is indicated on the left side: year of isolation, the hospital, type of ward, stage of CRKP pathogenesis (colonisation/infection), MLST type and if a mutation was present in previously-studied colistin resistance genes. Other acquired resistant genes are indicated in purple if they are present in the genome. If a resistance gene is truncated, it is represented in yellow. On the right section of the Figure, the pangenomes of the *K. pneumoniae* strains are respectively represented, with on top a scale referring to the length of the pangenome. Each blue band represents a gene in a pangenome. Genes that are present in all pangenomes are toward the left of the section (core genome), whereas those toward the right, are not universally present in the pangenomes (accessory genome).

**Figure 3:**
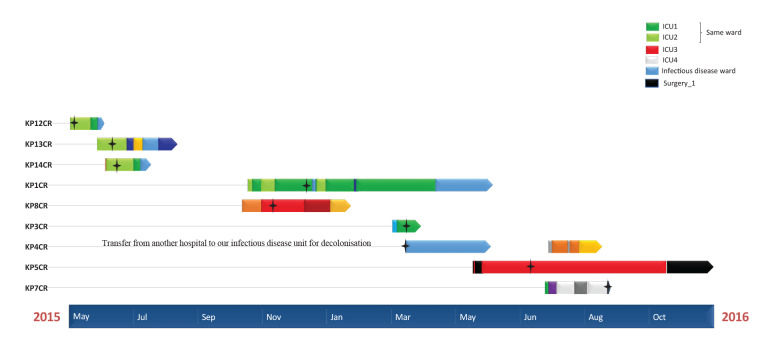
Time of hospitalisations in different wards of cases of *Klebsiella pneumoniae* ST307 resistant to colistin and time of the respective bacterial isolation, Marseille, France, 2015–2016 (n = 9 cases)

ST: sequence type; KP: *Klebsiella pneumoniae*; CR: colistin resistant.

Interestingly, five of the nine ST307 cases (KP12CR, KP13CR, KP14CR, KP1CR and KP3CR) met at different times between 2015 and 2016 in the same ward, i.e. intensive care unit (ICU)1 and ICU2 (Figure 3, light green and green) and became positive for CRKP in this ward. One additional case was present in the same ward (KP7CR) but never met the other CRKP positive cases. Another case (KP4CR), who had been previously hospitalised in a nearby town ICU and identified as a KPC carrier there, tested positive for KPCR on the first day of hospitalisation in Marseille. Interestingly, this person’s respective isolate and the isolates of the six other cases only presented between 2 and 13 SNPs (average 7 SNPs), confirming that all seven were from the same clone. However, two other cases (KP8CR and KP5CR) who were hospitalised in a common ward (ICU3, red), never met and genomes recovered from their isolates had between 91 and 114 different SNPs (average 102 SNPs), suggesting that they were affected by different strains. Moreover, these two strains were negative for the *bla_OXA48_* gene that was present in the other ST307 strains.

Each colour represents a different ward (only the wards of interest are described in the legend). The units in light green and green correspond to two units of the same ward; patients are often transferred between these two units according to the severity of their clinical conditions. The star indicates the first detection of a CRKP.

### Possible genetic basis of colistin resistance in *Klebsiella pneumoniae* strains

All strains were negative for the *mcr-1* to *mcr-8* gene variants. Mutations for the most common genes involved in colistin resistance were analysed (Table 3). All 14 strains analysed showed mutations in at least one colistin-resistance gene compared with the reference strain MGH78578. In nine of these, no mutation predicted or known to be responsible for colistin resistance was detected in any of the genes subjected to PROVEAN analysis.

**Table 3:** Summary of mutations found in the 14 strains of *Klebsiella pneumoniae* analysed, Marseille, France, 2014–2017 (n = 14 isolates)

Strain	ST	*wzi*	Colistin MIC (µg/mL)	PmrA	PmrB	PhoP	PhoQ	MgrB	CrrB	CrrA	AcrR	AcrS	*mcr*
KP1CR	307	NF^a^	16	A41T [13]	L213M [13]; T246A [26,37]	No^b^	No^b^	No^b^	C68S	No^b^	No^b^	S76R	No^b^
KP2CR	15	NF^a^	16	**G53V^c^** [26]	No^b^	No^b^	No^b^	No^b^	NF^a^	NF^a^	No^b^	No^b^	No^b^
KP3CR	307	NF^a^	16	A41T	L213M; T246A	No^b^	No^b^	No^b^	C68S	No^b^	No^b^	S76R	No^b^
KP4CR	307	173	8	A41T	L213M; T246A	No^b^	No^b^	No^b^	C68S	No^b^	No^b^	S76R	No^b^
KP5CR	307	173	16	A41T	L213M; T246A	**L12Q**	No^b^	No^b^	C68S	No^b^	No^b^	S76R	No^b^
KP6CR	15	NF^a^	16	No^b^	No^b^	No^b^	**L87P**	No^b^	NF^a^	NF^a^	No^b^	No^b^	No^b^
KP7CR	307	173	16	A41T	L213M; T246A	No^b^	No^b^	No^b^	C68S	No^b^	No^b^	S76R	No^b^
KP8CR	307	173	32	A41T	L213M; T246A	No^b^	No^b^	**Stop (13AA)** ^d^	C68S	No^b^	No^b^	S76R	No^b^
KP11CR	101	137	16	A217V	T246A	No^b^	No^b^	No^b^	NF^a^	NF	No^b^	H79Q	No^b^
KP12CR	307	173	8	A41T	L213M; T246A	No^b^	No^b^	No^b^	C68S	No^b^	No^b^	S76R	No^b^
KP13CR	307	173	16	A41T	L213M; T246A	No^b^	No^b^	No^b^	C68S	No^b^	No^b^	S76R	No^b^
KP14CR	307	173	4	A41T	L213M; T246A	No^b^	No^b^	No^b^	C68S	No^b^	No^b^	S76R	No^b^
KP15CR	322	50	> 64	S64T [26]	No^b^	No^b^	No^b^	**IS10 in bp 76** [38]	C68S	No^b^	No^b^	P211S	No^b^
KP16CR	101	137	4	A217V [13]	T246A	No^b^	N135K	No^b^	NF^a^	NF^a^	No^b^	H79Q	No^b^

AA: amino acid; CR: colistin resistant; IS: Insertion Sequence; KP:* Klebsiella pneumoniae*; MIC: minimum inhibitory concentration; NF: not found^a^; PROVEAN: Protein Variation Effect Analyzer; ST: sequence type.

^a ^The gene was not found.

^b^ No mutation was found in the gene compared with the reference strain MGH78578.

^c^ A mutation at this position was previously reported to alter protein function, but the substitution was G53C.

^d^ Stop codon at the codon for the 13^th^ AA, leading to a truncated protein.

Mutations in bold were predicted to modify protein function by PROVEAN analysis. Studies that have previously reported some of the mutations listed in the Table are respectively cited next to the mutation.

Five isolates (35.7%) had mutations in the *pmrA*, *phoP*, *phoQ* or *mgrB* genes and these mutations were predicted to modify protein function according to PROVEAN analysis (Table 3). We also noticed five mutations (PmrA A41T, PmrB L213M and T246A, CrrB C68S, AcrS S76R) that were present in all ST307 isolates. These were predicted by PROVEAN to not alter protein function independently. To explore if they might have, together, a synergistic role in colistin resistance and/or if they might be specific for ST307, we investigated the frequency of these mutations in 6,412 genomes of *K. pneumoniae *available on NCBI. Interestingly, simultaneous presence of the five mutations was found in 195 of 6,412 genomes analysed, including all 192 ST307 present in GenBank, one ST2739 (that belongs to CC307), one ST2975 and one genome with an unknown ST (Supplementary Figure S1). The frequency of the five mutations was strongly associated with the ST307 (192/195 vs 3/6,217; p < 0.001). Unfortunately, colistin susceptibility data were mostly lacking from the metadata of these genomes, preventing any insight into any potential association with colistin resistance.

Taken one by one, the frequency of each mutation in the 6,412 genomes was as follows: 3.2% (n = 204) of A41T in PmrA, 69.8% (n = 4,474) of C68S in CrrB and 3.3% (n = 209) of S76R in AcrS (Supplementary Table S5). A total of 288 genomes (4.5%) had mutations leading to presence of both L213M and T246A in the PmrB protein.

In terms of the occurrence of each ST or CC among the 6,412 genomes in GenBank, CC258 was most prevalent (n = 1,411; 22%), followed by ST11 (n = 715; 11%), ST15 (n = 336; 5%), ST101 (n = 206; 3%) and ST307 (n = 192; 3%) (Supplementary Figure S1). 

## Discussion

In this study, we retrospectively found 22 CRKP cases in four Marseille university hospitals. Univariate analysis identified several characteristics associated with carriage or infection with CRKP including male sex, length of hospital stay, recent bacterial infection, contact with a patient positive for CRKP and previous antibiotic therapy with fluoroquinolones, aminoglycosides and carbapenems. Furthermore, previous isolation of a carbapenemase-producing bacteria in patients was also determined as a risk factor, suggesting that controlling carbapenem resistance may possibly help mitigate emergence of CRKP in this setting.

In multivariate analysis, however, only male sex and contact with another patient carrying a CRKP remained as independent risk factors for CRKP acquisition. Therefore management options for patients carrying or infected by a CRKP strain could include isolation by trained staff, decolonisation and/or possibly faecal microbiota transplantation. Interestingly, in terms of patient care, a large proportion of the strains from the CRKP cases analysed here, were susceptible to common antibiotics that have been used in the past (e.g. mecillinam, nitrofurantoin, fosfomycin) [2]. Such antibiotics could represent valuable treatment alternatives in cases of CRKP infection.

The results of our case–control analysis confirmed those of other studies (Supplementary Table S6), though we did not identify colistin administration as a risk factor for CRKP acquisition. It should be nevertheless noted, that in our study, only five patients received colistin (two in the CSKP group vs three in the CRKP group), resulting in relatively weak statistical power.

Comparison between groups of cases and controls showed no difference in mortality. This finding differs from a few other previous investigations, which uncovered an excess mortality rate in cases of CRKP infections [7,24]; however, these studies focused on colistin-resistant carbapenemase-producing *K. pneumoniae* strains. Moreover, in our study, patients in both groups had various comorbidities that could have been confounding.

The *K. pneumoniae* isolates retrospectively considered for this study were selected based on colistin resistance only, without prior knowledge of their type. Of the 14 CRKP cases’ isolates, where sequencing was possible, nine were ST307, with seven of these belonging to the same clone. The two CRKP cases respectively bearing separate ST307 clones, never met. Among the seven cases affected by the same ST307 clone, five were hospitalised in the same ICUs at different – but sometimes overlapping – times between 2015 and 2016. As has already been demonstrated in colistin-resistance events [25], this might suggest cross-transmission. In this event, cross-transmission could have occurred in the ICU(s) via healthcare workers and/or environmental contamination. For the two remaining single-clone-affected CRKP cases, one had stayed in the same ICU as the other five, but at a completely different time, never meeting them. The other was never hospitalised in ICU, but was admitted to an infectious disease ward after transfer from another hospital near Marseille. This might indicate that the ST307 strain could present as an epidemic colistin-resistant clone, which has spread in our area, especially in the hospital environment [9,10]. In this regard, a national survey in France in 2014 detected three isolates with ST307 CRKP originating from the region Provence-Alpes-Côte d'Azur, to which Marseille belongs [8]. A French study in 2018 showed ST307 emerging in other areas of France, reinforcing its relevance [12].

Resistance to colistin in *K. pneumoniae* has previously been linked to several mechanisms including capsule overexpression, modifications of lipid A by addition of aminoarabinose or phosphoethanolamine due to mutations of target chromosomal genes or acquisition of plasmid-borne colistin resistance *mcr* genes [1]. All strains in our study were negative for *mcr* genes. However, among the 14 isolates analysed by WGS, substitutions, deletions and insertions in genes known to play a role colistin resistance, and likely to result in functional change at the protein level according to PROVEAN, were identified in five strains (two ST15, two ST307 and one ST322). In two of these (ST322 and ST15), the *mgrB* gene was inactivated by an insertion sequence (IS10) and by a mutation leading to a stop codon respectively, while in another (ST307), a substitution occurred at position 53 of PmrA (Table 3). Remarkably, mutations in MgrB have previously been reported to lead to a high MIC of colistin [1,26] as well as a mutation at position 53 in PmrA [26]. The two other mutations (PhoP L12Q and PhoQ P87L), which were respectively found in one ST307 and one ST15 strain, have never been described before and in vitro tests should be performed to confirm their role in colistin resistance.

Unfortunately, for the moment, we were not able to clearly decipher the molecular mechanism of colistin resistance for the remaining nine strains, two of which were ST101 and seven ST307. Of the latter, it can nevertheless be noted that five (i.e. KP4CR, KP7CR, KP12CR, KP13CR and KP14CR) had identical mutations compared to the reference strain MGH78578, but exhibited various colistin MICs. A different level of gene expression might explain these MIC differences, and this could be explored by transcriptomic analysis in further work. The mutations present in these five ST307 isolates (A41T in PmrA, L213M and T246A in PmrB, S76R in AcrS and C68S in CrrB) were also present in all nine ST307 sequences recovered from our CRKP cases. Subsequent finding that all 192 ST307 bacterial genomes in NCBI, as at April 2019, also shared these mutations was surprising. Unfortunately, colistin susceptibility data are mostly lacking from the metadata of these genomes, preventing insight into the relation of these mutations with colistin resistance. Further work is needed to study any possible association (Supplementary Table S7).

Interestingly, seven of the nine ST307 from our CRKP cases had the *wzi*-173 allele which seems to be associated with this ST [11] and which confers an additional capsular locus that may distinguish this particular phenotype [1].

During the 29^th^ European Congress of Clinical Microbiology and Infectious Diseases (ECCMID) in Amsterdam, the Netherlands (13–16 April 2019), ST307 was reported as an emerging clone in several countries including Italy [27,28], Russia [29], Qatar [30], France [31], South Africa [32], Spain [33] and the Netherlands [34]. From these reports, colistin resistance was found in 15 of 30 ST307 isolates detected in the Italian study and 24 of 35 isolates in the Russian one. Moreover, among a collection of 27 colistin-resistant *K. pneumoniae* from Kentucky, US [35], three were ST307. This raises the question as to whether our ST307 isolates from Marseille, France, are intrinsically resistant to colistin or if there is a propensity of ST307 clone to be or to become resistant to colistin everywhere in the world. 

We previously reported a case of an osteitis caused by a ST307 CSKP [36] treated with colistin, highlighting that this lineage was already present in our hospital centre in 2015. This isolate (Genbank accession number: NJGM01) had the same mutations in PmrA (A41T), PmrB (L213M and T216A) and AcrR (S76R) but the CrrAB proteins were absent. A SNP analysis between this colistin susceptible isolate and the colistin-resistant ST307 isolates from the current study (used as references) found between 995 and 1,546 SNPs (mean = 1,333) on an average of 5,540,016 bp analysed. The ST307 CRKP could have evolved from a CSKP clone in our setting. An exhaustive analysis of a larger sample collected from different sources would confirm this hypothesis.

This study has some limitations. The first is related to the retrospective nature of the study, which limited access to some clinical and laboratory data, such as the results from screening the patient for multidrug-resistant bacteria upon admission to hospital. The restrospective nature also impacted the availability of strains for sequencing from patients included in the study. Moreover as our initial aim was to molecularly characterise CRKP, no strains from patients in the CSKP control group for the case–control analysis were sequenced. The ST characterisation in this group might have provided more information on any relation between ST307 and the ‘colistin-resistant’ phenotype. The previous description of a carbapenemase-producing but colistin-susceptible *K. pneumoniae* ST307 strain suggests, however, that different clones of ST307 have diffused in our hospital, at least one susceptible and some resistant to colistin. Finally, as seven patients carried the same clone, this could have influenced the results of risk-factor analysis if there had been a clone–host relationship, such as a propensity of the clone to infect the same type of tissue or patient, however we did not observe this in our study.

In conclusion, this study concurs with previous ones, on risk factors for acquisition of CRKP and documents a CRKP ST307 epidemic clone in hospitals in Marseille, France, which is a country with an overall low prevalence of colistin resistance. Further work is warranted to understand why some clones have an ability to become increasingly resistant to antibiotics, by plasmid or chromosomal mechanism, as appears to be the case for CC258 [10] and ST307. 

## Supplementary Data

33000 Supplementary Dataset

## Supplementary Data

368000 Supplement
