# Supplementary material for: Risk factors for acquisition of colistin-resistant Klebsiella pneumoniae and expansion of a colistin-resistant ST307 epidemic clone in hospitals in Marseille, France, 2014 to 2017
Source: Euro Surveill. 2021 May 27;26(21):2000022. doi: 10.2807/1560-7917.ES.2021.26.21.2000022 (PMC8161728; doi:10.2807/1560-7917.ES.2021.26.21.2000022)

Supplementary material

This supplementary material is hosted by *Eurosurveillance* as supporting information alongside the article *Risk factors for acquisition of colistin-resistant* Klebsiella pneumoniae *and expansion of a colistin-resistant ST307 epidemic clone in hospitals in Marseille, France, 2014 to 2017* on behalf of the authors who remain responsible for the accuracy and appropriateness of the content. The same standards for ethics, copyright, attributions and permissions as for the article apply. Supplements are not edited by *Eurosurveillance* and the journal is not responsible for the maintenance of any links or email addresses provided therein.

Content:

Table S1. Genome accession numbers of the strains sequenced in this study

Table S2. Main features of *K. pneumoniae* ST307 genomes sequenced by Illumina Technology after A5-Assembly.

Table S3. Main features of other *K. pneumoniae* genomes sequenced by Illumina Technology after A5-Assembly.

Table S4. Treatment scheme of patients who received colistin during the study period.

Table S6. Summary of previous risk factors for colistin-resistance acquisition identified in literature.

Figure S1. a. Genotypes of genes of interest found in our ST307 genomes and frequencies of these genotypes in the 6,412 genomes available on PubMed (last upgrade April 2019). b. Repartition of the different STs in 6,412 genomes available on PubMed. The ST in red are the ones for which the previous mutations have been found.

Table S1. Genome accession numbers of the strains sequenced in this study.

Strain	Genome accession number
KP1CR	SEUA00000000
KP2CR	SETZ00000000
KP3CR	SETY00000000
KP4CR	SETX00000000
KP5CR	SETW00000000
KP6CR	SETV00000000
KP7CR	SETU00000000
KP8CR	SETT00000000
KP11CR	SETS00000000
KP12CR	SETR00000000
KP13CR	SETQ00000000
KP14CR	SETP00000000
KP15CR	SETO00000000
KP16CR	SETN00000000

Table S2. Main features of *K. pneumoniae* ST307 genomes sequenced by Illumina Technology after A5-Assembly.

Strain	KP1CR	KP3CR	KP4CR	KP5CR	KP7CR	KP8CR	KP12CR	KP13CR	KP14CR
**Demographical features**									
**Age of patient (year)**	42	78	89	75	79	77	55	55	75
**Sex**	M	M	M	F	M	M	M	M	M
**Year of isolation**	2015	2016	2016	2016	2016	2015	2015	2015	2015
**Nature of sample**	Rectal swab	Rectal swab	Rectal swab	Rectal swab	Urine	Drain liquid	BA	Puncture liquid	BA
**Unit**	ICU1	ICU1	Med2	ICU3	ICU4	ICU3	ICU2	ICU2	ICU2
**Antimicrobial susceptibility (mm)**									
**Piperacillin**	R	R	R	R	R	R	R	R	R
**Amoxicillin + clavulanic acid**	R	R	R	R	R	R	R	R	R
**Ticarcillin + clavulanic acid**	R	R	R	R	R	R	R	R	R
**Piperacilline + tazobactam**	R	R	R	I	R	I	R	R	R
**Cefoxitin**	R	I	I	R	I	S	I	R	R
**Ceftriaxone**	R	R	R	R	R	R	R	R	R
**Ceftazidime**	R	R	R	R	R	R	R	R	R
**Cefepim**	R	R	R	R	R	R	R	R	R
**Ertapenem**	R	R	R	R	R	S	R	R	R
**Meropenem**	I	I	I	I	I	S	S	S	S
**Imipenem**	S (2)[a]	S (0,5)[a]	S (1)[a]	S (1)[a]	S (1,5)[a]	S (0,38)[a]	S (1,5)[a]	S (2)[a]	S (1)[a]
**Aztreonam**	R	R	R	R	R	R	R	R	R
**Mecillinam**	R	S	S	R	S	S	S	S	S
**Ceftolozane/ tazobactam**	R (3)[a]	R (256)[a]	R (24)[a]	R (3)[a]	R (192)[a]	S (0,75)[a]	R (24)[a]	R (24)[a]	R (24)[a]
**Gentamicin**	R	R	R	R	R	R	R	R	R
**Amikacin**	S	S	S	S	S	S	S	S	S
**Tobramycin**	R	R	R	R	R	R	R	R	R
**Streptomycin**	R	R	R	R	R	R	R	S	S
**Spectinomycin**	S[b]	S[b]	S[b]	S[b]	S[b]	S[b]	S[b]	S[b]	S[b]
**Ciprofloxacin**	R	R	R	R	R	R	R	R	R
**Cotrimoxazole**	R	R	R	R	R	R	R	S	S
**Sulfadiazine**	R	R	R	R	R	R	R	S	S
**Fosfomycin**	R	S	S	R	S	S	S	S	S
**Nitrofurantoin**	S	S	S	R	S	S	S	S	S
**Doxycycline**	R (64)[a]	R (192)[a]	R (96)[a]	R (48)[a]	R (64)[a]	R (48)[a]	R (96)[a]	R (96)[a]	R (48)[a]
**Tigecycline**	R (4)[a]	I (2)[a]	I (2)[a]	S (1)[a]	R (3)[a]	S (1)[a]	I (2)[a]	I (2)[a]	I (2)[a]

**Minocycline**	R	R	R	I	R	S	R	R	R
**Rifampin**	R	R	R	R	R	R	R	R	R
**Chloramphenicol**	R (12)	R (16)	R (12)	S (4)	R (16)	S (2)	R (12)	R (12)	S (8)
**Colistin (MIC)**	16	16	16	16	16	32	8	16	4
**Genome features**									
**Size (bp)**	5,541,469	5,550,505	5,554,744	5,533,929	5,553,307	5,575,046	5,486,977	5,545,844	5,518,326
**G+C%**	57.2	57.2	57.2	57.2	57.2	57.3	57.2	57.2	57.2
**Median coverage**	34	55	42	50	42	39	48	25	47
**Number of contig**	133	84	83	75	81	196	78	96	74
**Number of rna**	86 tRNA, 10 rRNA, 1 tmRNA, 128 misc RNA	85 tRNA, 11 rRNA, 1 tmRNA, 127 misc RNA	82 tRNA, 11 rRNA, 1 tmRNA, 126 misc RNA	86 tRNA, 11 rRNA, 1 tmRNA, 125 misc_RNA	85 tRNA, 11 rRNA, 1 tmRNA, 128 misc RNA	85 tRNA, 11 rRNA, 1 tmRNA, 125 misc_RNA	83 tRNA, 9 rRNA, 1 tmRNA, 128 misc RNA	84 tRNA, 11 rRNA, 1 tmRNA, 126 misc RNA	85 tRNA, 12 rRNA, 1 tmRNA, 128 misc RNA
**Number of CDS**	5,150	5,166	5,167	5,120	5,168	5,165	5,097	5,162	5,135
**ST**	307	307	307	307	307	307	307	307	307
wzi	NTy	NTy	173	173	173	173	173	173	173
**Resistome**									
blaOXA-1	+	+	+	+	+	+	+	+	+
blaSHV-28-1	+	+	+	+	+	+	+	+	+
blaTEM-1	-	+	+	-	+	+	+	+	-
blaCTX-M-15	+	+	+	+	+	+	+	-	+
blaOXA-48	+	+	+	-	+	-	+	+	+
StrB	+	-	-	-	-	-	-	-	-
aac(3)-IIa_1	+	+	+	+	+	+	+	+	+
aac(6')Ib-cr_1	+	+	+	+	+	+	+	+	+
aph(3'')-Ib_5	+	+	+	+	+	+	+	+	-
aph(6)-Id_1	-	+	+	+	+	+	+	+	-
QnrB1	+	+	+	+	+	+	+	+	+
SulII	+	+	+	+	+	+	+	+	
DfrA	+	-	+	+	+	-	+	+	+
tetA	+	+	+	+	+	+	+	+	+
catB4	+	+	+	+	+	+	+	+	+
**Plasmid replicon**									
Inc L/M	+	+	+	-	+	-	+	+	+
IncFIB	+	+	+	+	+	+	+	+	+

BA: Bronchial aspiration; ICU: Intensive care unit; Med: Medical services; NT: not tested; NTy: Non-typable.

[a] Minimal Inhibitory Concentration (MIC) in mg/L.

[b] The chosen diameter cut-off for interpretation is those of *Neisseria gonorrheae* which present an identical ECOFF with *Enterobacteriaceae* in MIC (mg/L)

Table S3. Main features of other *K. pneumoniae* genomes sequenced by Illumina Technology after A5-Assembly.

Strain	KP2CR	KP6CR	KP11CR	KP16CR	KP15CR
**Demographical features**					
**Age of patient (year)**	69	65	1	73	1 month
**Sex**	M	M	M	F	F
**Year of isolation**	2016	2016	2014	2017	2017
**Nature of sample**	Blood	Rectal swab	Urine	Urine	Pus
**Unit**	Med1	ICU3	Surg1	Surg2	NICU1
**Antimicrobial susceptibility**					
**Piperacillin**	R	R	R	R	S
**Amoxicillin + clavulanic acid**	R	R	R	R	S
**Ticarcillin + clavulanic acid**	R	R	R	R	S
**Piperacilline + tazobactam**	I	R	R	S	S
**Cefoxitin**	S	R	R	S	S
**Ceftriaxone**	R	R	R	R	S
**Ceftazidime**	R	R	R	R	S
**Cefepim**	R	R	R	R	S
**Ertapenem**	S	R	R	S	S
**Meropenem**	S	S	R	S	S
**Imipenem**	S (0,25)[a]	S (0,5)[a]	R (12)[a]	S (0,38)[a]	S (0,19)[a]
**Aztreonam**	R	R	R	R	S
**Mecillinam**	S	R	S	S	S
**Ceftolozane/ tazobactam**	S (0,75)[a]	S (0,75)[a]	R (256)[a]	R (3)[a]	S (0,25)[a]
**Gentamicin**	S	R	R	R	S
**Amikacin**	S	S	R	R	S
**Tobramycin**	R	R	R	R	S
**Streptomycin**	I	S	R	R	S
**Spectinomycin**	R[b]	R[b]	R[b]	R[b]	S[b]
**Ciprofloxacin**	R	R	R	R	S
**Cotrimoxazole**	R	R	R	R	S
**Sulfadiazine**	R	R	R	R	S
**Fosfomycin**	S	R	R	R	S
**Nitrofurantoin**	S	S	S	R	S
**Doxycycline**	S (1.5)[a]	I (6)[a]	R (256)[a]	R(>256)[a]	R (12)[a]
**Tigecycline**	S (0.19)[a]	S (0.5)[a]	I (2)[a]	S (0.5)[a]	S (0.5)[a]
**Minocycline**	S	S	R	R	I
**Rifampin**	R	R	R	R	R
**Chloramphenicol**	S (1.5)[a]	S (3)[a]	S (8)[a]	S (4)[a]	S (1)[a]
**Colistin (MIC)**	16	6	16	4	>64

**Genome features**					
**Size (bp)**	5,666,043	6,002,255	5,912,443	5,653,198	5,410,915
**G+C%**	57.1	56.7	56.3	57	57.3
**Median coverage**	43	41	19	42	44
**Number of contig**	98	289	603	98	94
**Number of rna**	85 tRNA, 11 rRNA, 1 tmRNA, 132 misc_RNA	84 tRNA, 11 rRNA, 1 tmRNA, 128 misc_RNA	93 tRNA, 19 rRNA, 1 tmRNA, 137 misc RNA	85 tRNA, 13 rRNA, 1 tmRNA, 129 misc RNA	83 tRNA, 12 rRNA, 1 tmRNA, 137 misc_RNA
**Number of CDS**	5,276	5,617	5,422	5,322	5,046
**ST**	15	15	101	101	322
***wzi* gene**	NTy	NTy	137	137	50
**Resistome**					
blaDHA-1		+			
blaOXA-1	+	+	+	+	
blaOXA-9			+	+	
blaSHV-1-15			+	+	
blaSHV-11					+
blaSHV-28-1	+	+			
blaTEM-1	+	+		+	
blaCTX-M-15	+	+	+	+	
blaOXA-48			+		
aac(3)-IIa_1		+	+	+	
aac(6')-Ib			+		
aac(6')Ib-cr_1	+	+			
aadA2	+	+			
aph(3')-Ia	+	+			
aph(3'')-Ib_5				+	
aph(6)-Id_1				+	
QnrB1		+			
QnrB4		+			
Sul1	+	+			
SulII				+	
DfrA	+	+	+	+	
tetA				+	
tetB					+
tetD			+	+	
catB4	+	+	+	+	
mph(A)_2		+			
**Plasmid replicon**					
IncHI2		+			
Inc L/M			+		
IncFIB	+	+		+	
IncR_1			+	+	
ColRNAI_1			+		

ICU: Intensive care unit; NICU: Neonates intensive care unit; Med: Medical services Surg: Surgery services; NT: not tested; NTy: Non-typable.

[a] Minimal Inhibitory Concentration (MIC) in mg/L.
[b] The chosen diameter cut-off for interpretation is those of *Neisseria gonorrheae* which present an identical ECOFF with *Enterobacteriaceae* in MIC (mg/L)

Table S4. Treatment scheme of patients who received colistin during the study period.

Patient	Colistin susceptibility	Type of infection	Monotherapy/ association	Route of administration	Posology	Treatment duration (days)	Clinical outcome
KP19CR	Resistant	Acute pyelonephritis	Association	IV	3 MUI x 3	7	Death
KP21CR	Resistant	Osteoarticular	Vancomycin + colistin	IV	NA	300 days	Alive
KP22CR	Resistant	VAP	Colistin + meropenem	IV + aerosol	4.5 MUI x 2	7	Death
KP14	Susceptible	VAP	Monotherapy	Aerosol	NA	27	Alive
KP60	Susceptible	VAP	Monotherapy	Aerosol	NA	3	Alive

VAP: Ventilator associated pneumonia.

Table S6. Summary of previous risk factors for colistin-resistance acquisition identified in literature.

Type of statistical analysis		Risk factor	Bacterial species	Reference
Multivariate and univariate analysis		Use of colistin	GNB	1–8
		Male sex	GNB	9
		Number of CR-KPC positive patients in nearby beds per day	*K. pneumoniae*	3
		Corticosteroid administration	*K. pneumoniae*	3
		Previous KPC-KP colonization	*K. pneumoniae*	3,4
		>3 previous hospitalizations	*K. pneumoniae,* GNB	4,10
		Charlson score>3	*K. pneumoniae*	4
		Neutropenia	*K. pneumoniae*	4,9
		Previous carbapenem administration	*K. pneumoniae*-GNB	6–9
		Age	*K. pneumoniae*	6
		Beta-lactams/ Beta-lactams inhibitor exposure	GNB	7
		Prior use of fluoroquinolone	GNB	9
		Non-independent functional capacity	GNB	7
		Hospital stay >2 weeks	GNB	10
		Exposure to >3 antibiotic classes	GNB	10
		Abnormality/surgery of the lower urinary tract	GNB	10
Univariate analysis	Demographics	Age	GNB	1,6
		Male sex	GNB	5
		Duration of intensive care unit stay	GNB	1,2
		Number of chronic disease	*K. pneumoniae*	3
		Previous hospitalization >1 or >2	*K. pneumoniae*	4,6
		Admission from another healthcare facilities	*K. pneumoniae*	4,5,11
		Charlson morbidity index	GNB	7
		Elixhauser score	*K. pneumoniae*	5
	ICU procedures	In ICU at the time of culture	*K. pneumoniae*	5
		Any ICU stay during index hospitalization	*K. pneumoniae,* GNB	5,8
		Recent bacterial infections	*K. pneumoniae*	4
		Duration of mechanical ventilation	*K. pneumoniae*	1,5
		Tracheotomy	*K. pneumoniae,* GNB	3,5,8
		Number of invasive catheter	*K. pneumoniae,* GNB	3,8,11
		Presence of indwelling urinary catheter	*K. pneumoniae*	5
		Parenteral nutrition	*K. pneumoniae*	3
		Surgical procedures	GNB	1
		Nasogastric tube	GNB	8
	Antibiotic administration	Number of antibiotics administrated	*K. pneumoniae*	3
		Prior use of antifungals	GNB	1
		Duration of use of third-generation cephalosporins	*K. pneumoniae*	2,7
		Use of β-lactam β-lactamase inhibitor	*K. pneumoniae*	4,7
		Mean duration of use of β-lactam β-lactamase inhibitor	*K. pneumoniae*	11

		Prior use of carbapenems	*K. pneumoniae*	[3,5,6,8,9,12]
		Prior use of aminoglycosides	*K. pneumoniae*	[3,7]
		Prior use of Tigecycline	*K. pneumoniae*	[3]
		Prior use of Linezolid	*K. pneumoniae*	[3]
		Prior use of Glycopeptide	*K. pneumoniae*	[6]
		Mean antibiotic use per day	*K. pneumoniae*	[3,7]
		Median length of colistin treatment	*K. pneumoniae*	[2]
	Colonization pressure	Current isolate is carbapenem-resistant	*K. pneumoniae*	[5]
		Prior isolation of carbapenem-resistant GNR	*K. pneumoniae*	[5]
		Days at risk	*K. pneumoniae,* GNB	[3,8]
		Number of CR-KPC positive patients in ICU per day	*K. pneumoniae*	[2]
	Labs on day of culture	WBC	*K. pneumoniae*	[5]
		Hemoglobin	*K. pneumoniae*	
		Hematocrit	*K. pneumoniae*	
		BUN	*K. pneumoniae*	
		GFR	*K. pneumoniae*	
		Alkaline Phosphatase	*K. pneumoniae*	
	Days since	Last antibiotic	*K. pneumoniae*	
		Last Aminoglycoside	*K. pneumoniae*	
		Last Anti-pseudomonal carbapenem	*K. pneumoniae*	
		Last dose of ertapenem	*K. pneumoniae*	
		Last Penicillin	*K. pneumoniae*	
		Last anti-MRSA	*K. pneumoniae*	
		Last colistin	*K. pneumoniae*	
		Last carbapenem (any)	*K. pneumoniae*	
		Last beta-lactam	*K. pneumoniae*	
		Last Probiotic	*K. pneumoniae*	

GNB: Gram-negative bacteria; KPC-Kp: *K. pneumoniae* producing KPC enzyme.; CR-KPC: Colistin-resistant carbapenemase producing *K. pneumoniae.*

1 Matthaiou DK, Michalopoulos A, Rafailidis PI, *et al.* Risk factors associated with the isolation of colistin-resistant gram-negative bacteria: a matched case-control study. *Crit Care Med* 2008; **36**: 807–11.

2 Kontopidou F, Plachouras D, Papadomichelakis E, *et al.* Colonization and infection by colistin-resistant Gram-negative bacteria in a cohort of critically ill patients. *Clin Microbiol Infect* 2011; **17**: E9–11.

3 Papadimitriou-Olivgeris M, Christofidou M, Fligou F, *et al.* The role of colonization pressure in the dissemination of colistin or tigecycline resistant KPC-producing *Klebsiella pneumoniae* in critically ill patients. *Infection* 2014; **42**: 883–90.

4 Giacobbe DR, Del Bono V, Trecarichi EM, *et al.* Risk factors for bloodstream infections due to colistin-resistant KPC-producing *Klebsiella pneumoniae*: results from a multicenter case-control-control study. *Clin Microbiol Infect* 2015; **21**: 1106.e1-8.

5 Richter SE, Miller L, Uslan DZ, *et al.* Risk Factors for Colistin Resistance among Gram-Negative Rods and *Klebsiella pneumoniae* Isolates. *J Clin Microbiol* 2018; **56**. DOI:10.1128/JCM.00149-18.

6 Gundogdu A, Ulu-Kilic A, Kilic H, *et al.* Could Frequent Carbapenem Use Be a Risk Factor for Colistin Resistance? *Microb Drug Resist* 2018; **24**: 774–81.

7 Drozdinsky G, Ben-Zvi H, Kushnir S, Leibovici L, Yahav D. Colistin exposure as a risk factor for infections caused by inherently colistin resistant Enterobacteriaceae-a case-control study. *Clin Microbiol Infect* 2018; **24**: 896–9.

8 Teo JQ-M, Chang CW-T, Leck H, *et al.* Risk factors and outcomes associated with the isolation of polymyxin B and carbapenem-resistant Enterobacteriaceae spp.: A case-control study. *Int J Antimicrob Agents* 2019; **53**: 657–62.

9 Wang Y, Tian G-B, Zhang R, *et al.* Prevalence, risk factors, outcomes, and molecular epidemiology of mcr-1-positive Enterobacteriaceae in patients and healthy adults from China: an epidemiological and clinical study. *Lancet Infect Dis* 2017; **17**: 390–9.

10 Kaza P, Mahindroo J, Veeraraghavan B, Mavuduru RS, Mohan B, Taneja N. Evaluation of risk factors for colistin resistance among uropathogenic isolates of *Escherichia coli* and *Klebsiella pneumoniae*: a case-control study. *J Med Microbiol* 2019; **68**: 837–47.

11 Zarkotou O, Pournaras S, Voulgari E, *et al.* Risk factors and outcomes associated with acquisition of colistin-resistant KPC-producing *Klebsiella pneumoniae*: a matched case-control study. *J Clin Microbiol* 2010; **48**: 2271–4.

12 Büchler AC, Gehringer C, Widmer AF, Egli A, Tschudin-Sutter S. Risk factors for colistin-resistant Enterobacteriaceae in a low-endemicity setting for carbapenem resistance - a matched case-control study. *Euro Surveill* 2018; **23**. DOI:10.2807/1560-7917.ES.2018.23.30.1700777.

13 Page AJ, Cummins CA, Hunt M, *et al.* Roary: rapid large-scale prokaryote pan genome analysis. *Bioinformatics* 2015; **31**: 3691–3.

Figure S1. a. Genotypes of genes of interest found in our ST307 genomes and frequencies of these genotypes in the 6,412 genomes available on PubMed (last upgrade April 2019). b. Repartition of the different STs in 6,412 genomes available on PubMed. The ST in red are the ones for which the previous mutations have been found.

a.

Gene	**PmrA**	**PmrB**	**CrrA**	**CrrB**	**AcrR**	**AcrS**	**Total**
Genotype	A41T	L213M T246A	wild	C68S	wild	S76R	All
Number of genomes with the genotype	204	288	6,031	4,474	6,164	209	195
Number of genomes without the genotype	6,208	6,124	381	1,938	248	6,203	6,217

b.

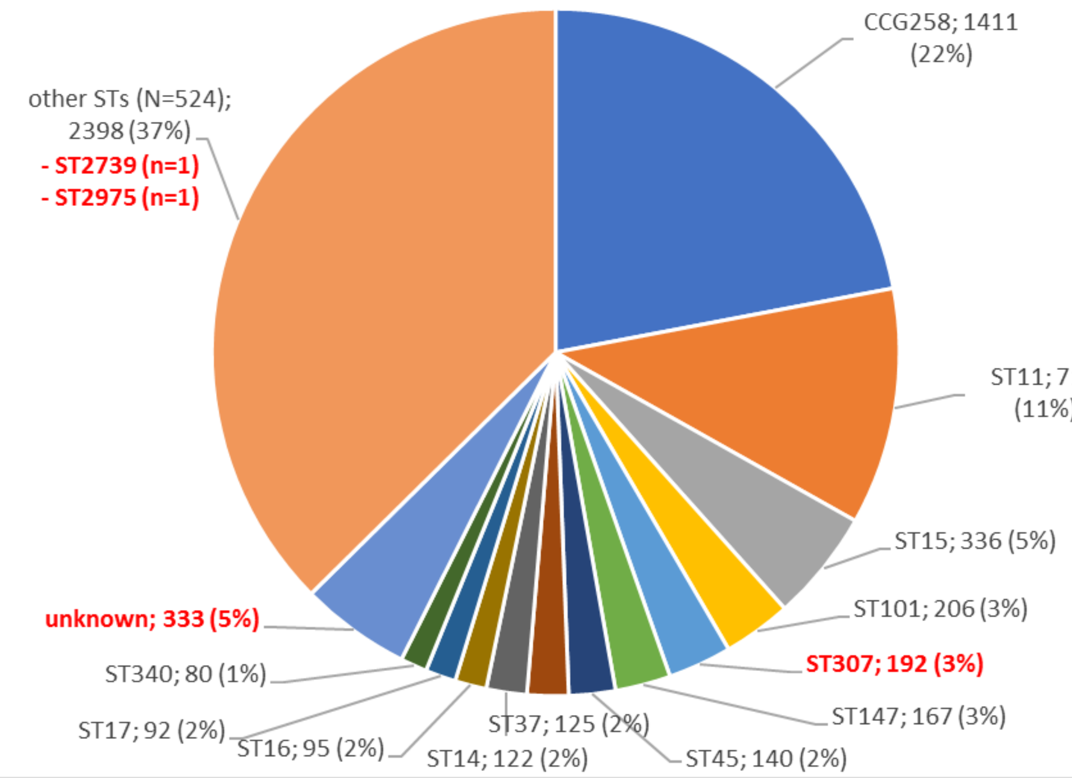

Supplement: Supplement [file 2000022_Supplementaryfiles.pdf]
